# Intrinsic androgen-dependent gene expression patterns revealed by comparison of genital fibroblasts from normal males and individuals with complete and partial androgen insensitivity syndrome

**DOI:** 10.1186/1471-2164-8-376

**Published:** 2007-10-18

**Authors:** Paul-Martin Holterhus, Uta Deppe, Ralf Werner, Annette Richter-Unruh, Jan-Hendrik Bebermeier, Lutz Wünsch, Susanne Krege, Hans-Udo Schweikert, Janos Demeter, Felix Riepe, Olaf Hiort, James D Brooks

**Affiliations:** 1 Department of Pediatrics, University-Hospital Schleswig-Holstein, Campus Kiel, Schwanenweg 20, Kiel, Germany; 2 Department of Pediatric and Adolescent Medicine, University-Hospital Schleswig-Holstein, Campus Lübeck, Ratzeburger Allee 160, Lübeck, Germany; 3 Endokrinologikum Ruhr, Alter Markt 4, Bochum, Germany; 4 Department of Pediatric Surgery, University Hospital Schleswig-Holstein, Campus Lübeck, Ratzeburger Allee 160, Lübeck, Germany; 5 Department of Urology, University of Essen, Essen, Germany; 6 Department of Internal Medicine, University of Bonn, Bonn, Germany; 7 Department of Genetics, Stanford University School of Medicine, CA, USA; 8 Department of Urology, Stanford University School of Medicine, CA, USA

## Abstract

**Background:**

To better understand the molecular programs of normal and abnormal genital development, clear-cut definition of androgen-dependent gene expression patterns, without the influence of genotype (46, XX vs. 46, XY), is warranted. Previously, we have identified global gene expression profiles in genital-derived fibroblasts that differ between 46, XY males and 46, XY females with complete androgen insensitivity syndrome (CAIS) due to inactivating mutations of the androgen receptor (AR). While these differences could be due to cell autonomous changes in gene expression induced by androgen programming, recent work suggests they could also be influenced by the location from which the fibroblasts were harvested (topology). To minimize the influence of topology, we compared gene expression patterns of fibroblasts derived from identical urogenital anlagen: the scrotum in normally virilized 46, XY males and the labia majora from completely feminized 46, XY individuals with CAIS.

**Results:**

612 transcripts representing 440 unique genes differed significantly in expression levels between scrotum and CAIS labia majora, suggesting the effects of androgen programming. While some genes coincided with those we had identified previously (TBX3, IGFBP5, EGFR, CSPG2), a significant number did not, implying that topology had influenced gene expression in our previous experiments. Supervised clustering of gene expression data derived from a large set of fibroblast cultures from individuals with partial AIS revealed that the new, topology controlled data set better classified the specimens.

**Conclusion:**

Inactivating mutations of the AR, in themselves, appear to induce lasting changes in gene expression in cultured fibroblasts, independent of topology and genotype. Genes identified are likely to be relevant candidates to decipher androgen-dependent normal and abnormal genital development.

## Background

Androgen receptor (AR) signaling is the key determinant of virilization in male external genitalia development [1-4]. Its importance is highlighted in the androgen insensitivity syndrome (AIS), a virtual human AR "knock-out" due to inactivating mutations of the AR gene, and characterized by defects in virilization of 46, XY individuals despite normal or elevated serum testosterone levels. The phenotype can range from normal female in complete AIS (CAIS), to lesser degrees of genital ambiguity in partial AIS (PAIS) in which the degree of virilization is related to the degree of AR function [2,3]. Genital masculinization involves a comprehensive re-organization of genital anatomy in which androgens induce a permanent male developmental fate in the originally bipotent anlagen. For example, in response to embryonal androgen signaling, the labioscrotal swellings will develop into a scrotum and not into labia majora (as overviewed by [5]). These observations indicate the existence of androgen-mediated gene expression programs that are responsible for implementation and persistence of male-specific genital morphology and function. In general, distinguishing X- and Y-chromosomal influences from hormonal influences on genital development has proven difficult [6,7]; however, CAIS, in which all individuals possess an XY genotype, represents the ideal situation for focusing on the role of androgens.

In a previous study, we reported evidence for androgen-mediated programming of gene expression by comparing genital skin fibroblasts from normal males to those derived from CAIS individuals [8]. However, the demonstration that cultured fibroblasts show stable transcription signatures that reflect their site of origin in mature adult tissues (transcriptional topographic memory) [9] raises the possibility that fibroblast transcript signatures might also reflect androgen-independent aspects of their developmental history. Were that the case, the transcript signatures we had identified might be influenced both by androgen signaling during genital development and topology. Even though the normal male and CAIS fibroblasts were both derived from the genital skin, their precise sites of biopsy differed both topologically and with regard to embryological origin. The normal male genital fibroblasts were grown from biopsies of the foreskin (derived from the genital tubercle), while CAIS fibroblasts came from biopsies of the labia majora (derived from the labioscrotal swellings) [8]. To better define the gene expression programs dependent only on developmental androgen actions, we analyzed gene expression profiles of fibroblasts derived from homologous embryonic structures: the labioscrotal swellings in 46, XY males (scrotum) and 46, XY females (labia majora). We validated the biological relevance of this approach by comparing the ability of this new gene expression set and the previous set to classify a large set of individuals representing the whole clinical spectrum of AIS phenotypes based on gene expression profiles alone.

## Results

### Identification of a topology-independent, AR-dependent gene expression program

**Figure 1:**
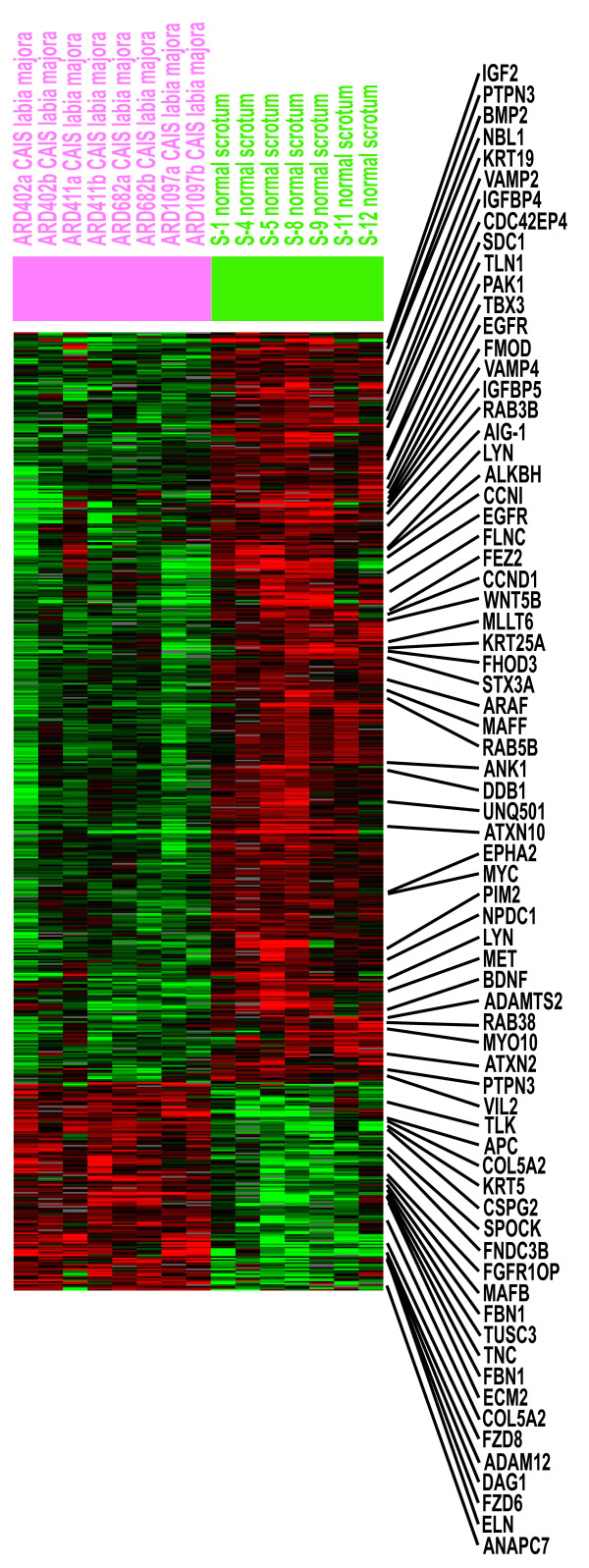
**Transcripts with significant differences of expression levels between normal scrotum and CAIS labia majora**. Transcript levels of 612 genes identified by SAM analysis as differing between fibroblasts derived from normal male scrotum (green) and labia majora of 46, XY females with CAIS (pink). Individual transcripts are grouped by hierarchical cluster analysis and are displayed in rows while experiments are represented in columns. Expression values per gene are centered by the mean log_2 _red/green normalized ratio. Increasing red intensity corresponds to higher relative transcript levels compared to the mean expression level across all 15 array experiments. Increasing green intensity corresponds to relatively decreased transcript levels compared to the mean. On the right side, examples of individual genes (gene symbols according to S.O.U.R.C.E. [24]) discussed in the paper or falling into the biological processes and cellular pathways detected by PANTHER are displayed. Detailed data on figure 1 is available in additional files 1, 2, 3, 4.

We had previously identified an AR-dependent gene expression signature by comparing normal male foreskin fibroblasts to those cultured from diverse sites in the genitals of patients with documented CAIS. Subsequent reports that cultured fibroblasts retain topographic transcriptional memory (gene expression signatures that reflect their site of biopsy) led to concerns that the AR-dependent gene expression signature we identified could have been affected by topological differences in the fibroblast samples used. To test for this potential confounder, we repeated microarray experiments on seven independent strains of normal male scrotal fibroblasts (S1, S4, S5, S8, S9, S11, and S12) and duplicate samples of four labia majora fibroblasts derived from 46, XY individuals with CAIS due to proven inactivating mutations of the AR-gene. Both fibroblasts were derived from identical anlagen: the labioscrotal swellings. The SAM procedure revealed 612 transcripts representing 440 unique genes that differed significantly in expression level between the groups at a false discovery rate of 0.038 (Figure 1).

The new topology-controlled data set showed some similarities to the AR-dependent gene set we had identified previously, with 42 unique transcripts found in both gene sets, including 34 transcripts that were up-regulated in normal male derived fibroblasts, and 8 up-regulated in the CAIS female-derived fibroblasts. Genes up-regulated in normal male fibroblasts in both data sets included TBX3 (T-box 3), CBX6 (Chromobox homologue 6), IGFBP5 (Insulin-like growth factor binding protein 5), and EGFR (epidermal growth factor receptor) while several others were no longer identified as significantly different between the data sets, such as TBX2 (T-box 2), TBX5 (T-box 5), BMP4 (bone morphogenetic protein 4), HOXA13 (Homeobox A13), WNT2 (Wingless-type MMTV integration site family, member 2), and FOXF2 (Forkheadbox F2). The significant change in the gene lists strongly suggested that topology influenced the gene expression signatures identified in our original series of experiments.

### Topology independent AR gene expression program classifies diverse AIS samples

**Figure 2:**
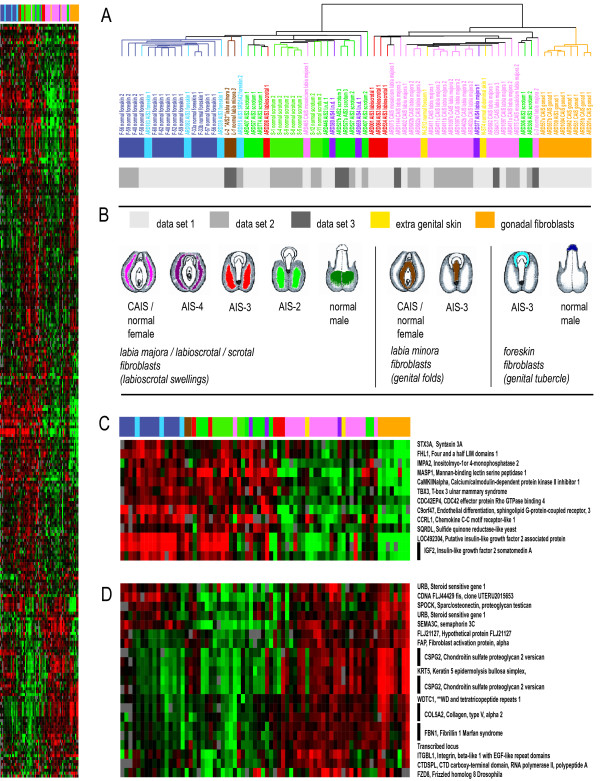
**Cluster analysis of normal male fibroblasts from scrotum and foreskin as well as 46, XY individuals with PAIS and CAIS**. Hierarchical clustering analysis of 72 microarray experiments of cultured genital fibroblasts using the SAM derived gene list. The heatmap on the left displays 259 genes that had at least 85% interpretable data across the experiments whose expression levels were at least 2-fold different from the mean expression across all samples in at least 5 microarrays. (A) The cluster dendrogram demonstrating the degree of relatedness (Pearson correlation) between the expression patterns of the 259 genes in the cultured fibroblast samples. The length of the arms of the dendrogram reflects the degree of correlation between the samples. Samples are color coded to reflect the localization of the biopsy and the degree of external genital virilization according to a grading scheme developed by Sinnecker et al. [10]. The grey bar below indicates whether a sample was derived from dataset 1, 2 or 3. ''L.n.d.'' signifies that the biopsy localization was not accurately documented. Italics indicate a sample with a 46, XX karyotype. (B) Schematic depiction of the external genitalia phenotype of the cases from which the fibroblast cultures were derived using color coding that corresponds to the degree of genital ambiguity and the location of the biopsy. Color coding corresponds to the bar below the dendrogram in (A). (C) Cluster of AR-dependent transcripts that are highly expressed in the left ''male'' major branch of the cluster that are expressed at significantly lower levels in the ''female'' branch of the cluster on the right. TBX3, previously reported in ulnar mammary syndrome and IGF2, previously reported as being down-regulated in CAIS [20] are shown in this cluster. (D) Cluster of AR-dependent transcripts that are expressed at significantly lower levels in the lefthand ''male'' branch of the cluster and at higher levels in the righthand ''female'' branch. These include many extracellular matrix genes such as proteoglycan testican, versican, and fibrillin 1. Detailed data on figure 2 is available in additional files 5, 6, 7, 8.

To evaluate the relevance of the topology-controlled, AR dependent gene list, we tested its ability to classify 72 microarray experiments performed on fibroblast samples derived from 51 individuals that included normal males, normal females, and individuals with PAIS and CAIS (Fig. 2 and table 1). AIS samples were graded according to the system suggested by Sinnecker [10] wherein phenotypically male genitalia are scored AIS 1, while female external genitalia are scored AIS 5 (Fig. 2B). Stringent filtering conditions of the combined data sets reduced the number of transcripts from 612 to 259; however, altering the stringency of the filtering conditions and the number of transcripts used did not significantly change the clustering pattern of the individual samples (data not shown).

**Table 1:** Fibroblast strains

**cell strain**	**micro-array data set**	**anatomic origin *embryonal anlagen***	**external genitalia *AIS-stage***	**age at biopsy years; months**	**selected functional data (androgen binding, AR-genotype, specific remarks)**
**F-33**	1	foreskin *genital tubercle*	normal fertile male	51;11	K_d _0.08 nM, B_max _26 fmol/mg protein
**F-40**	1, 2	foreskin *genital tubercle*	normal male	7;11	K_d _0.09 nM, B_max _32 fmol/mg protein
**F-52**	1	foreskin *genital tubercle*	normal male	0;3	K_d _0.06 nM, B_max _35 fmol/mg protein
**F-56**	1, 2	foreskin *genital tubercle*	normal fertile male	42;10	K_d _0.07 nM, B_max _13 fmol/mg protein
**F-57**	1	foreskin *genital tubercle*	normal male	5;11	K_d _0.05 nM, B_max _24 fmol/mg protein
**F-58**	1	foreskin *genital tubercle*	normal male	5;11	K_d _0.10 nM, B_max _31 fmol/mg protein
**F-59**	1, 2	foreskin *genital tubercle*	normal male	0;8	K_d _0.07 nM, B_max _40 fmol/mg protein
**F-60**	1	foreskin *genital tubercle*	normal male	5;4	K_d _0.05 nM, B_max _20 fmol/mg protein
**F-62**	1	foreskin *genital tubercle*	normal male	2;0	K_d _0.08 nM, B_max _86 fmol/mg protein
**ARD339**	1	foreskin *genital tubercle*	ambiguous **(AIS3)**	1;4	Glu798Gln
**ARD082**	1	foreskin *genital tubercle*	ambiguous **(AIS3)**	10;11	Asp604Tyr
**ARD183**	1	foreskin *genital tubercle*	ambiguous **(AIS3)**	5;1	Asp756Ser
**ARD515**	2	foreskin *genital tubercle*	ambiguous **(AIS3)**	0;6	homozygous missense mutation Arg227Gln in SRD5A2 gene leading to 5α reductase type II deficiency
**S-1**	2	scrotum *labioscrotal swellings*	normal male	30;0	no functional data
**S-4**	2	scrotum *labioscrotal swellings*	normal male	1;3	K_d _0.12 nM, B_max _44 fmol/mg protein
**S-5**	2	scrotum *labioscrotal swellings*	normal male	1;7	K_d _0.09 nM, B_max _28 fmol/mg protein
**S-8**	2	scrotum *labioscrotal swellings*	normal male	31;0	no functional data
**S-9**	2	scrotum *labioscrotal swellings*	normal male	34;0	K_d _0.08 nM, B_max _55 fmol/mg protein
**S-11**	2	scrotum *labioscrotal swellings*	normal male	32;2	K_d _0.08 nM, B_max _26 fmol/mg protein
**S-12**	2	scrotum *labioscrotal swellings*	normal male	9;2	K_d _0.07 nM, B_max _37.09 fmol/mg protein
**ARD527**	1, 2, 3	scrotum *labioscrotal swellings*	predominantly male **(AIS2)**	1;6	Leu712Phe, K_d _0.21 nM; B_max _38 fmol/mg protein
**ARD446**	1	scrotum *labioscrotal swellings*	predominantly male **(AIS2)**	7;8	silent mutation GGG>GGA in codon 795 of the AR-gene K_d _0.07 nM, B_max _26 fmol/mg protein
**ARD407**	1, 2	scrotum *labioscrotal swellings*	predominantly male **(AIS2)**	1:0	polyglycin repeat of the N-terminus reduced to 10 repeats plus Ala645Asp, K_d _0.09 nM; B_max _36 fmol/mg protein
**ARD306**	1, 2	scrotum *labioscrotal swellings*	predominantly male **(AIS2)**	7;8	Arg855His; K_d _0.97 nM, B_max _20 fmol/mg protein
**ARD774**	1	scrotum *labioscrotal swellings*	predominantly male **(AIS2)**	0;11	Ala596Thr; K_d _0.08 nM; B_max _43 fmol/mg protein
**ARD208**	1	labia majora/scrotum *labioscrotal swellings*	ambiguous **(AIS3)**	1;1	Val746Met; K_d _0.16 nM; B_max _3 fmol/mg protein
**ARD534**	1	labia majora/scrotum *labioscrotal swellings*	ambiguous **(AIS3)**	2;4	Arg608Lys
**ARD084**	1	labia majora/scrotum *labioscrotal swellings*	ambiguous **(AIS3)**	0;8	no mutation in the whole coding region of the AR gene, reduced AR-mRNA, reduced AR-protein and reduced ligand binding (K_d _0.05 nM; B_max _6 fmol/mg protein)
**ARD001**	1	labia majora/scrotum *labioscrotal swellings*	ambiguous **(AIS3)**	0;9	exonic splice site mutation AGC>AGT in codon 888 leading to aberrant splicing
**ARD380**	1	labia majora/scrotum *labioscrotal swellings*	ambiguous **(AIS3)**	13;1	Arg615Pro, post zygotic mutation leading to somatic mosaicism
**ARD377**	1	labia majora/scrotum *labioscrotal swellings*	predominantly female **(AIS4)**	1;2	Ile841Ser, (K_d _0.55 nM; B_max _17 fmol/mg protein),
**ARD659**	1	labia majora *labioscrotal swellings*	Predominantly female **(AIS4) **	3;10	Ala870Gly
**ARD1097**	1, 2	labia majora *labioscrotal swellings*	normal female **(CAIS)**	1;3	Pro390Ser + Arg855Gly, negative androgen binding
**ARD411**	1, 2	labia majora *labioscrotal swellings*	normal female **(CAIS)**	0;4	Arg855Cys, negative androgen binding
**ARD1144**	3	labia majora *labioscrotal swellings*	normal female **(CAIS)**	4;3	pathological androgen binding, K_d _1.59 nM, B_max _14 fmol/mg protein, no mutation detected
**GS641-7/ARD291**	3	labia majora *labioscrotal swellings*	normal female **(CAIS)**	18;4	Phe794Ser, negative androgen binding
**ARD682**	1, 2	labia majora *labioscrotal swellings*	normal female **(CAIS)**	14;10	Gln59stop, negative androgen binding, no AR-protein in Western immunoblot
**ARD402**	1, 2	labia majora *labioscrotal swellings*	normal female **(CAIS)**	1;0	2 base pair deletion in exon 1, frameshift, premature stop codon, negative androgen binding, very low AR-mRNA transcription, no AR-protein in Western immunoblot
**ARD075**	1, 2	labia majora *labioscrotal swellings*	normal female **(CAIS)**	12;3	Arg774Cys, post zygotic mutation leading to somatic mosaicism, K_d1 _0.03 nM; B_max1 _2 fmol/mg protein (wilde type AR); K_d2 _8.5 nM; B_max2 _29 fmol/mg protein (mutant AR)
**ARD465**	1	labia majora *labioscrotal swellings*	normal female **(CAIS)**	5;5	Glu287stop, low expression of wild-type AR (K_d _0.11 nM; B_max _4 fmol/mg protein), post-zygotic mutation (somatic mosaicism likely)
**L-1**	3	labia minora*urethral folds*	normal female	37;10	normal female (46, XX)
**L-2**	3	labia minora*urethral folds*	normal female	23;0	female (46 XX), classical congenital adrenal hyperplasia due to 21-hydroxylase deficiency, external genital virilization of Prader 3 corresponding phenotypically to "AIS3"
**L-3**	3	labia majora *labioscrotal swellings*	normal female	40;4	normal female (46, XX)
**ARD709**	1	testis *gonad*	ambiguous **(AIS3)**	6;5	Met775Ile
**ARD291**	1	testis *gonad*	normal female **(CAIS)**	18;4	Phe794Ser, negative androgen binding
**ARD531**	1	testis *gonad*	normal female **(CAIS)**	35,2	Ala765Thr, negative androgen binding
**ARD557**	1	testis *gonad*	normal female **(CAIS)**	6;6	donor splice site exon 2/intron 2, negative androgen binding
**ARD842**	1	testis *gonad*	normal female **(CAIS)**	38;0	26 bp deletion exon 1 (141–150), frameshift, premature stop codon, negative androgen binding
**ARD1004**	1	testis *gonad*	normal female **(CAIS)**	17;4	Val866Met, negative androgen binding
**N-ST4**	1	abdominal skin	normal male	46;0	no functional data
**N-LS12**	1	forearm skin	normal male	36;0	no functional data

Fibroblast strains; microarray dataset in whichh they have been analysed (1 – 3); ARD or GS, patient strain-ID; F, normal male foreskin fibroblasts; S, normal male scrotal fibroblasts. L-1-3 represent the only samples with a 46, XX karyotype. N-ST4 and N-LS12 are of extragenital origin. ARD515 was derived from a 46, XY male with ambiguous genitalia due to a 5α reductase type II defect rather than AIS. Normal ranges for androgen (methyltrienolone) binding: B_max _(binding capacity): 13–116 fmol/mg protein; K_d _(dissociation constant): 0.03–0.13 nM.

Clustering separated the 72 experiments into two major subgroups. The righthand major branch included predominantly patients with female external genitalia while most of the patients in the lefthand major branch had normal male or highly virilized external genitalia (Fig. 2A and 2B). All but one of the samples with CAIS (AIS 5) and a normal 46, XX female clustered in the right ("female") branch (Fig. 2A). Interestingly, the one exception expressed a wild type AR in a portion of cells due to somatic mosaicism (ARD465). Two skin fibroblast samples from normal males derived from regions without an obvious androgen induced sexual dimorphism (abdomen, forearm) also clustered in the right ("female") branch. The left ("male") major branch contained all genital skin fibroblasts derived from normal male controls and 8 of 10 microarry experiments reflecting patients with higher degrees of virilization due to partial AIS (AIS 2). This cluster also included a fibroblast sample from an individual with 5α-reductase type II deficiency, a defect which results in ambiguous genitalia due to lack of conversion of testosterone to dihydrotestosterone. This individual presumably possesses a wild-type AR, meaning that androgen signaling pathways remained intact. Three of the four labioscrotal fibroblast samples from individuals with AIS 3 phenotypes with significant genital ambiguity clustered in the "female" major branch and the remaining in the "male" one (Fig. 2). Of note, clustering did not appear to be influenced by array type or RNA reference-type, indicating that normalization procedures did not influence data quality.

Structure within the cluster dendrogram suggested that there was some residual influence of topology on gene expression in the samples. In the righthand "female" branch, fibroblast samples derived from AIS gonads clustered separately from all skin-derived samples (Fig. 2A). Similarly, the lefthand "male" branch showed a subcluster that contained all the foreskin-derived fibroblasts, including the AIS 2 fibroblasts originating from the foreskin. Interestingly, this branch also contained two strains of labia minora fibroblasts from two 46, XX individuals, one of whom had ambiguous genitalia (Prader stage 3) due to 21-hydroxylase deficiency (female pseudohermaphroditism), while the other individual was a normal female. Since the labia minora are analogous to the urethral folds that participate in penile morphogenesis, this finding suggests that topographic origin influenced expression within the selected set of genes of these two samples more than AR signaling. In some cases, the anatomic origin of biopsy was not well documented (table 1). This might explain why some samples did not cluster as expected (e.g., ARD380 and ARD659, Fig. 2A) although other factors might have contributed to these findings.

**Figure 3:**
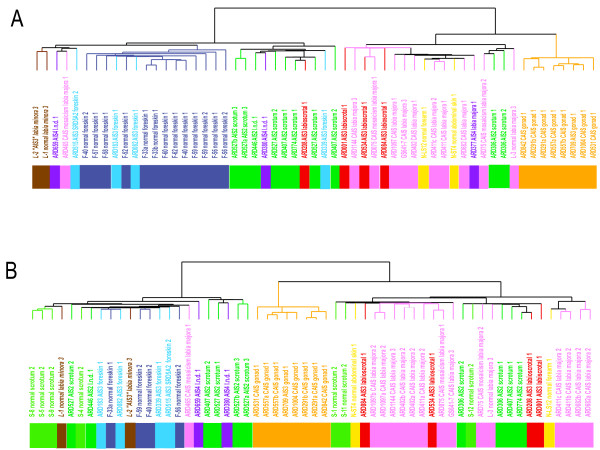
**Experiment clustering with topography controlled (A) – versus previous (B) [8] gene set**. Hierarchical clustering analyses of microarray experiments using the new topology-independent AR gene list (A) and our previous gene list [8] that did not control for topology of biopsy location (B). In each of the two clusters A and B, the microarray experiments used to generate the underlying list of significant genes by SAM were removed before clustering resulting 57 remaining experiments in (A) and 58 in (B), respectively. The color code is the same as in Fig. 2. Based on the previous gene set, several highly virilized AIS 2 patients and normal scrotal fibroblasts were incorrectly classified female.

We also wanted to reconsider whether the new topology-independent, AR gene list better classified samples than our previous gene list that did not control for the locations from which samples were harvested. After removing the microarray experiments that were used to define either the previous [8] or the new gene set by SAM, the remaining samples were clustered. As expected, in case of both gene lists the samples sorted into two main branches that separated primarily male and female samples (Fig. 3A and 3B). Moreover, when we considered only topology-controlled samples originating from the labioscrotal swellings excluding the mosaic samples and those of insufficiently described biopsy localization, the mean AIS-grades between the two branches differed significantly using both gene lists (new gene list: AIS-grades: 2.5 ± 0.55 (male); 4.17 ± 1.12 (female); p < 0.001 by t-test; previous gene list (Holterhus et al. 2003): AIS-grades: 1.6 + 0.79 (male); 3.3 + 1.6 (female); p < 0.01 by t-test). However, in contrast to the new gene set, the previous gene set that did not account for topology misclassified many individuals. It resulted in incorrectly female classification of most of the highly virilized individuals with AIS 2 (3 of 4 individuals = 75%) and of a large fraction of the normal male scrotal fibroblast controls (3 of 7 individuals = 43%) (Fig. 3A and 3B). The new gene set misclassified only one individual with AIS 2 (ARD306).

### Biological processes in the AR-dependent gene expression program

We performed a systematic analysis for enrichment of genes belonging to defined biological processes and cellular pathways using the PANTHER classification system [11]. PANTHER classifies genes by their functions, based on published experimental evidence and on evolutionary relationships. The 612 significant transcripts corresponded to 527 named transcripts, of which PANTHER recognized 440 unique gene IDs. Several related biological processes were significantly over-represented in the AR-dependent gene list including "control of cell proliferation and differentiation" (p = 0.00001, "developmental processes" (p = 0.00013) and "cell cycle control" (p = 0.00041) (table 2). Analysis of cellular pathways also revealed several interesting signaling pathways including "angiogenesis" (p = 0.00001) and WNT-signaling" (p = 0.00002) (table 3). These processes and pathways were reflected in the major branches of the cluster dendrogram revealing differential expression in the phenotypically male and female samples (Figures 2C and 2D). For instance, samples in the "male" branch showed high expression of CCN1 (cyclin 1), CCND1 (cyclin D1), IGF2 (insulin-like growth factor 2), IGFBP5 (Insulin-like growth factor binding protein 5), MYC (V-myc myelocytomatosis viral oncogene homolog), MAFF (V-maff musculoaponeurotic fibrosarcoma oncogene homolog F), EGFR (epidermal growth factor receptor), PTPN3 (protein tyrosine phophatase), MET (hepatocyte growth factor receptor) and several other genes important in cell growth and proliferation (Fig. 1 and 2A). Transcripts expressed at high levels in the "female" branch included ANAPC7 (anaphase promoting complex subunit 7), FZD8 (frizzled homolog 8) and FZD6 (frizzled homolog 6) (Fig. 1).

**Table 2:** Biological processes

**Biological process**	**Detected number of genes in SAM list**	**Expected number of genes based on NCBI reference list**	**P-value**
**Cell proliferation and differentiation**	40	18.46	0.00001
**Intracellular signaling cascade**	35	15.59	0.00001
**Protein phosphorylation**	28	12.07	0.00005
**Protein modification**	40	20.81	0.00008
**Developmental processes**	61	37.57	0.00013
**Biological process unclassified**	157	193.59	0.00024
**Cell cycle control**	18	7.16	0.00041
**Cell structure and motility**	36	20.29	0.00076
**Signal transduction**	86	61.52	0.0008
**Extracellular matrix protein-mediated signaling**	6	1.09	0.00091

AR-dependent *biological processes *identified by PANTHER (Thomas et al. 2003) [11] as significantly over-represented in the SAM-list compared to the NCBI human reference gene list (table limited to genes with p < 0.001)

**Table 3:** Cellular pathways

**Cellular pathway**	**Detected number of genes in SAM-list**	**Expected number of genes based on NCBI reference list**	**P-value**
**Angiogenesis**	16	4.12	0.00001
**Wnt signaling**	20	6.71	0.00002
**T cell activation**	10	2.26	0.00012
**Inflammation mediated by chemokine and cytokine signaling pathway**	16	5.9	0.00038
**Endothelin signaling pathway**	8	1.84	0.00145
**Muscarinic acetylcholine receptor 1 and 3 signaling pathway**	5	0.81	0.00145
**Integrin signaling pathway**	12	4.44	0.00202
**Alpha adrenergic receptor signaling pathway**	4	0.53	0.00209
**EGF receptor signaling pathway**	8	2.56	0.00470
**Unclassified**	370	388.65	0.00476
**Axon guidance mediated by semaphorins**	5	1.15	0.00636
**PDGF signaling pathway**	9	3.38	0.00781
**Ras pathway**	6	1.73	0.00852

AR-dependent *cellular pathways *identified by PANTHER (Thomas et al. 2003) [11] as significantly over-represented in the SAM-list compared to the NCBI human reference gene list (table limited to genes with p < 0.01)

The AR-dependent gene set showed enrichment for a number of genes related to maintenance and modification of tissue shape and structural identity (Tables 2 and 3, and Fig. 2D). Genes that were up-regulated in the predominately male branch were SDC1 (syndecan 1), FMOD (fibromodulin) and ADAMTS2 (A disintegrin-like and metalloproteinase with thrombospondin type 1 motif, 2). Those up-regulated mainly in the female branch included ADAM12 (A disintegrin and metalloproteinase 12), TNC (tenascin C), CSPG2 (chondroitin sulfate proteoglycan 2, versican), FBN1 (fibrillin 1), ELN (elastin), COL5A2 (collagen, type V, alpha2), ECM2 (extracellular matrix protein 2, female organ and adipocyte specific), DAG1 (dystroglycan 1), and SPOCK (sparc/osteonectin, cwcv kazal-like domains proteoglycan, testican).

### Confirmation of selected transcripts by RT-PCR

**Figure 4:**
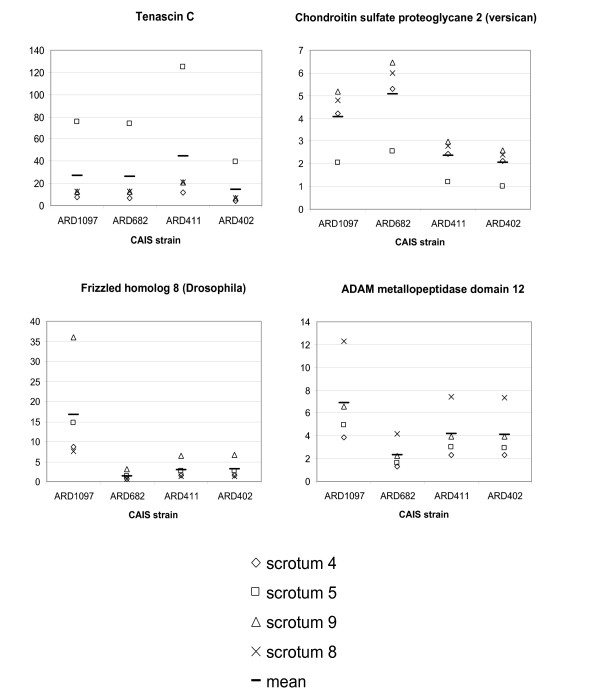
**Verification of selected genes by RT-PCR**. The ratio of transcript levels of TNC (Tenascin), FZD8 (Frizzled 8), ADAM12 (ADAM metallopeptidase 12), and CSPG2 (Chondroitin sulfate proteoglycane 2, versican) comparing CAIS cell lines and normal scrotal cell lines. Semi-quantitative RT-PCR was performed on four samples of normal male scrotal fibroblasts (S4, S5, S8, S9), as well as on four samples of labia majora derived from CAIS patients (ARD402, ARD411, ARD682, ARD1097). Differences in expression levels between different cell lines were calculated according to the ΔΔ-CT method [19]. The y-axis represents the ratios of expression levels of CAIS labia majora fibroblasts divided by those from scrotal fibroblasts.

Four transcripts were selected for confirmation of the microarray data by semi-quantitative RT-PCR: TNC (tenascin), FZD8 (frizzled 8), ADAM12 (ADAM metallopeptidase 12), and CSPG2 (chondroitin sulfate proteoglycane 2, versican). RNA from the labia majora fibroblasts derived from CAIS affected individuals (ARD402, ARD411, ARD682, ARD1097), as well as four samples of the normal male scrotal fibroblasts (S4, S5, S8, S9) were used for analysis. In agreement with the microarray data, TNC, FZD8, ADAM12 and CSPG2 show significantly higher expression in the labia majora fibroblasts compared to the scrotal fibroblasts (Fig. 4).

## Discussion

We demonstrate reproducible, lasting differences in gene expression in cultured fibroblasts harvested from analogous structures in the genital tissues of normal males and 46, XY CAIS females. This unique model system, in which samples were matched for genotype and topology, allows identification of gene expression programs predominantly influenced by AR signaling, likely during genital morphogenesis. By selecting fibroblasts derived from the labioscrotal swellings in normal and CAIS individuals, we were able to largely exclude androgen-*in*dependent mechanisms as a cause of systematic differences of baseline gene expression. While we had identified a related set of genes previously using fibroblast cells that had not been matched for site of origin, the significant differences between our previous and current gene sets implies that topology significantly influenced gene expression in our original set. This finding is instructive for future studies seeking to identify the effects of inactivating mutations of single genes in tissues or cells cultured from tissues. Gene expression profiles can be influenced by obvious genotypic differences, such as the presence of an X or Y chromosome [6] but also by subtle differences including differences in topology [9].

The transcripts we have identified appear to be highly relevant to male and female external genital morphogenesis. When used to classify an independent set of fibroblasts cultured from normal male, normal female, and PAIS-affected individuals, this gene set performed considerably better than our previous set in which we had not controlled for differences in topology. The observation that fibroblasts derived from the labia majora of normal female external genitalia clustered with AIS derived samples from the same site demonstrates the primacy of developmental androgen actions in influencing gene expression in this set of genes, and possibly in external genital morphogenesis. Furthermore, our findings suggest that gene expression profiling of genital fibroblasts might be correlated with phenotype in vivo and could serve as a diagnostic marker of the extent of developmental androgenization, although many more cases will need to be analyzed, and other analytic methods will need to be used to identify an ideal classifier.

Chang and co-workers have demonstrated that fibroblasts cultured from different regions of the body retain position-specific expression signatures [9]. Our work confirms the presence of lasting, topology-influenced differences in gene expression in human fibroblasts. Controlling for the site from which genital fibroblasts were derived significantly altered the set of genes we identified as differing between normal and CAIS-derived fibroblasts. Despite that control, signatures of topology remained in our new gene set. For instance, normal male fibroblasts and CAIS-derived gonadal fibroblasts each clustered as distinct entities in the "male" and "female" arms, respectively. Thus, even in our stringently selected fibroblast samples and gene set, topology remains evident in the gene expression profile. Although Chang and co-workers have found that HOX gene signatures account for much of the topological gene classifier, our gene list lacked HOX genes, implying that traces of topology remain encoded in other sets of transcripts. Together with the results of Chang et al., our work demonstrates substantial heterogeneity in gene expression in cultured fibroblasts that can be influenced by topology and, in the case of our unique fibroblast samples, AR signaling. We suspect that other signaling pathways, possibly including other steroid hormones or growth factors, can program lasting changes gene expression in fibroblasts. Whether these differences in gene expression can influence development of associated tissues in vivo remains to be elucidated. Regardless, our data suggests one possible model system for teasing out the effects of mutations in single genes on global gene expression patterns in cells cultured from in vivo models.

The biological processes and the cellular pathways enriched in the AR-dependent gene set could provide some insights into the role of AR-mediated programming of fibroblasts in male and female external genital differentiation. For instance, activation of several growth signaling pathways in male derived fibroblasts and WNT signaling pathways in female genital fibroblasts, e.g., FZD8 (Fig. 1, 2 and 4), might signal their important roles in genital development. The same could be true for genes contributing to maintenance and modification of tissue shape and structural identity, e.g., versican, tenascin, and ADAM12 (Fig. 1, 2 and 4). It is unclear, however, whether expression of these genes in cultured fibroblasts reflects their expression in vivo, whether expression is necessary for maintenance of external genital structure, or whether these pathways are reactivated as a result of cell culture. Moreover, it cannot be excluded that potentially higher cumulative estrogens during development and postnatal life in the AIS patients until the time of genital skin biopsy may have had additional influences on gene expression. It is possible that the gene expression programs have little to do with genital development; however, the finding that many genes related to cell and tissue structure and integrity is tantalizing. We identified many genes of the cytoskeletal network (KRT5, 19, 25A, MYO10) and of the surrounding extracellular matrix (TNC, SPOCK, CSPG2, COL5A2) in the AR-dependent gene expression data set [12-14]. Additional in vivo work including strategies comprising early embryonal urogenital tissues will clarify whether genes identified using our approach are important in external genital morphogenesis.

## Conclusion

Our data demonstrate the existence of large-scale AR-dependent gene expression programs in fibroblasts cultured from human external genitalia. These programs are influenced neither by differences in sex chromosomes nor by the topographic differences between the genital tubercle and the labioscrotal swellings. Improved classification of individuals with partial and complete androgen insensitivity syndrome with this gene list compared to a set we had identified previously suggests that androgen programming must have played a key role in establishing these programs. Therefore, the detected genes represent valuable targets for unraveling human external genital differentiation and the role of androgen therein.

## Methods

The study was approved by the ethical committee of the University of Lübeck, Germany. Informed consent was obtained from the control subjects, affected individuals or their parents.

### Cell strains

A total of 51 different primary fibroblast cultures were analyzed (table 1). Genital fibroblasts were established from scrotal skin biopsies from normal males undergoing orchidopexy or testis biopsy (scrotal fibroblasts) or from circumcisions (foreskin fibroblasts). Normal female genital skin fibroblasts were established from individuals undergoing genital plastic surgery (labia majora fibroblasts). Genital skin fibroblasts of patients with disorders of sex development (DSD, [15]), mostly AIS, were obtained either for diagnostic reasons or during reconstructive surgery (foreskin-derived or labioscrotal-derived or labial fibroblasts) or following gonadectomy (gonadal fibroblasts). Extra-genital skin fibroblasts were obtained from phenotypically normal male individuals. For gene expression profiling, fibroblasts of the 3rd to 7th passage were cultured to confluency (wherein they entered G_0 _arrest), as described previously [8].

### RNA-isolation and cDNA-labeling

Protocols for mRNA and total RNA preparation, and cDNA labeling are available at  [16]. Either 2 μg of mRNA (dataset 1) or 50 μg of total RNA (datasets 2 and 3) from genital fibroblasts were reverse transcribed and labeled with Cy5. Labeled cDNAs from genital fibroblasts were mixed with Cy-3-labeled reference RNA from Stategene (datasets 2 and 3) or a pooled reference of RNA from fibroblasts and 11 immortalized cell lines (dataset 1, see [8]).

### Microarrays and hybridizations

Poly L-lysine coated (data set 1 = 19 new hybridized micoarrays plus 22 previously published microarrays [8] and dataset 2 = 24 new hybridized microarrays) or Corning glass slide (data set 3 = 7 new hybridized microarrays) spotted cDNA-microarrays containing more than 44,000 elements corresponding to approximately 26,000 unique genes (UniGene clusters) were used for gene expression analysis (for details, see [16]). Hybridizations were performed using equal amounts of Cy3 and Cy5 labeled cDNAs according to previously published protocols for 14–18 hours over night at 65°C. Hybridized microarrays were scanned using a GenePix4000 array scanner and analyzed with GenePix Pro software (Axon Instruments, Inc., Union City, CA).

### Microarray data analysis

To identify genes differentially expressed in homologous tissues of normal male and CAIS female derived fibroblasts, we restricted our analysis to samples derived from the labioscrotal swellings (scrotum or labia majora), that were hybridized on poly-lysine coated slides against Strategene reference RNA (data set 2). Only spots with fluorescence signals of 1.5-fold over array background in either the experimental or reference channel were considered. The Cy5/Cy3 fluorescence ratios for all genes in each array were normalized to obtain an average absolute log_2 _red/green ratio of 0 and the fluorecence ratios for each gene were mean centered. The Significance Analysis of Microarrays (SAM) procedure [17] was used to identify transcripts with statistically significant differences in expression levels between normal and CAIS samples. Identified transcripts were used to perform hierarchical clustering analysis of 72 microarray experiments performed on fibroblasts derived from normal males, and individuals affected with PAIS and CAIS. To correct for differences in the reference RNA (dataset 1 versus dataset 2 and 3) and slide coating (dataset 1 and 2 versus dataset 3), normalized log_2 _red/green ratios for single genes were centered by the mean in each of the three datasets separately according to a previously published procedure [18]. Centered data from the three data sets were combined and again were mean-centered for each gene. In the combined dataset, only genes with measurable data in 85% of the 72 experiments were considered. Restriction of the analysis to genes which had absolute values of normalized log_2 _red/green ratios ≥ 1.0 in at least 5 experiments of the sample set of 72 microarrays resulted in 259 transcripts. Microarray experiments and genes were organized by hierarchical clustering, using the Pearson correlation metric and average linkage clustering [19]. TreeView software was used to visualize the results [19]. Supplementary information on each of the microarray experiments and all datasets are available online [22,23]. For figure 1 please see additional files 1, 2, 3, 4, for figure 2, please see additional files 5, 6, 7, 8.

### Functional classification of sexually dimorphic genes

SAM identified transcripts were loaded into the PANTHER (Protein ANalysis THrough Evolutionary Relationships) database. Enrichment of biological processes and cellular pathways in the AR dependent gene list was determined by comparing the number of genes observed in the SAM-list for each process or pathway to the number of genes expected based on the NCBI Homo sapiens reference gene list. PANTHER determines a p-value based on binominal statistics, describing the probability that the number of detected genes occurred randomly, with a p-value < 0.05 indicating significance [11]. Since many pathways can be identified, we focused our discussion on a subset in biological processes with p < 0.001 and in cellular pathways with p < 0.01.

### RT-PCR analyses

Semi-quantitative RT-PCR was performed using FAM (F) labeled probes with dabcyl (DB) quencher. RT-PCR was performed on four samples of normal male scrotal fibroblasts (S4, S5, S8, S9) as well as on four samples of labia majora derived from CAIS patients (ARD402, ARD411, ARD682, ARD1097). Exon-spanning primers and probes (TIB Molbiol, Berlin, Germany) had the following sequences: Tenascin: TNC-forward: ACAgTgggACAgCAggTgACT, Tm: 59.6°C; TNC-reverse: CAggTTgACACggTgACAgTTC, Tm: 59.4°C, TNC labeled probe: F-TACCACAATggCAgATCCTTCTCCACCT-DB, Tm: 66.7°C; Frizzled 8: FZD8-forward: gTgCAgCgAAgggACACTTg, Tm: 61.0°C; FZD8-reverse: TCCTCAgCCAACAgAAATTAACg, Tm: 58.7°C, FZD8 labeled probe: F-AggTTCCCACCCCTTCACAgTgTTgA-DB, Tm: 67.9°C; ADAM metallopeptidase 12: ADAM12-forward: CCTggCACCCCTCAgACC, Tm: 60.9°C, ADAM12-reverse: gTACAAAAAACTCCAACTggAgCTg, Tm: 59.4°C, ADAM12 labeled probe: F-TCCACACCAAgTgCCCAgATCCAC-DB, Tm: 67.0°C; Chondroitin sulfate proteoglycane 2, versican: CSPG2-forward: TgTCTCACgAAgAACAAATgTTTgT, Tm: 58.0°C, CSPG2-reverse: gggTCTCCAATTCTCgTATTgCA, Tm: 59.8°C, CSPG2 labeled probe: F-TgCCATCAgTCCAACggAAgTCATgCT-DB, Tm: 69.3°C; TATA-binding protein (TBP) was used to normalize expression values. TBP primers were TBP-forward: CACgAACCACggCACTg-ATT, TBP-reverse: TTTTCTTgCTgCCAgTCTggAC; TBP-labeled probe: F-TgTgCAC-AggAgCCAAgAgTgAAgA-DB. Reverse transcription was performed using the Ambion RETROscript kit according to the manufacturer's recommendations with minor modifications. 2 μg of total RNA, 0.5 μM dNTPs, 5 μM random decamers and 200 units of MMLV-RT polymerase were used per reaction. Heat denaturation was performed for 3 min at 85°C preceding reverse transcription for 1 hr at 44°C and subsequent inactivation for 10 min at 92°C. C-DNA corresponding to 12 ng of initial total RNA served as template for amplification reactions. 0.4 μM of each primer, 0.17 μM of specific probe and TaqMan Universal PCR mastermix (Applied Biosystems, Foster City, CA, USA) including the Taq polymerase were added to a final volume of 25 μl. Initial denaturation was performed at 95°C for 10 min followed by 1 min cycling intervals at 60°C using a RotorGene RG-3000 cycler (Corbett-Research, Sydney, Australia). Differential transcription levels between all different cell lines were calculated according to the ΔΔ-CT method [20].

## Abbreviations

AR, androgen receptor; AIS, androgen insensitivity syndrome; CAIS, complete androgen insensitivity syndrome; PAIS, partial androgen insensitivity syndrome

## Authors' contributions

All authors read and approved the final manuscript. PMH was responsible for the experimental design, assessment of clinical information on AIS-patients (including clinical examination), culturing fibroblasts, RNA-works and microarray hybridizations (data set 1), functional characterization of AR in fibroblast samples, data analysis, and he was the leading writer of the manuscript. UD planned and performed culturing fibroblasts, RNA-works, microarray hybridizations, microarray data analysis and management (data set 2 and 3), and contributed essentially to writing the manuscript. RW helped essentially with the microarray strategies and data analysis and contributed to writing the manuscript. He was also responsible for RT-PCR. ARU was responsible for clinical assessment of patients and contributed to writing the manuscript. In particular, she contributed the 46, XX fibroblasts. JHB performed microarray data analysis and wrote the manuscript. LW was responsible for clinical assessment of several patients, and he obtained many fibroblast biopsies, especially the normal scrotal controls, and contributed to writing the manuscript. SK assessed many patients clinically and contributed to writing the manuscript. HUS was reponsible for clinical assessment of several patients (data set 3), contributed functional characterization of AR in fibroblast samples (several of the androgen binding studies) and contributed to writing the manuscript. JD was indispensable for microarray data analysis, central data management at Stanford University and the web repository of data. He also helped in creating the figures for the web supplement. FR assessed many patients clinically, took part in data analysis and was one of the writers of the manuscript. OH was an important senior clinical supervisor of genital phenotype assessment, he contributed most of the molecular data of the androgen receptor gene to the study and he was a key person in data interpretation with respect to androgen insensitivity syndrome. JDB was the senior writer of the manuscript. He supervised the experimental strategy, including all statistical issues with respect to the microarray data.

## Supplementary Material

Additional file 1 Text file to visualize data for figure 1 together with additional files 2, 3 and 4 in TreeView [19]. Click here for file

Additional file 2 Cluster file to visualize data for figure 1 together with additional files 1, 3 and 4 in TreeView [19]. Click here for file

Additional file 3 Array tree file to visualize data for figure 1 together with additional files 1, 2 and 4 in TreeView [19]. Click here for file

Additional file 4 Gene tree file to visualize data for figure 1 together with additional files 1, 2 and 3 in TreeView [19]. Click here for file

Additional file 5 Text file to visualize data for figure 2 together with additional files 6, 7 and 8 in TreeView [19]. Click here for file

Additional file 6 Cluster file to visualize data for figure 2 together with additional files 5, 7 and 8 in TreeView [19]. Click here for file

Additional file 7 Array tree file to visualize data for figure 2 together with additional files 5, 6 and 8 in TreeView [19]. Click here for file

Additional file 8 Gene tree file to visualize data for figure 2 together with additional files 5, 6 and 7 in TreeView [19]. Click here for file

## References

[B1] Wilson JD, Griffin JE, Leshin M, George FW (1981). Role of gonadal hormones in development of the sexual phenotypes. Hum Genet.

[B2] Quigley CA, De Bellis A, Marschke KB, el-Awady MK, Wilson EM, French FS (1995). Androgen receptor defects: historical, clinical, and molecular perspectives. Endocr Rev.

[B3] Hiort O, Holterhus PM (2000). The molecular basis of male sexual differentiation. Eur J Endocrinol.

[B4] MacLaughlin DT, Donahoe PK (2004). Sex determination and differentiation. N Engl J Med.

[B5] Grumbach MM, Conte FA, Hughes IA, Larsen PR, Kronenberg HM, Melmed SM, Polonsky MS (2002). Disorders of Sex Differentiation. Williams Textbook of Endocrinology.

[B6] Whitney AR, Diehn M, Popper SJ, Alizadeh AA, Boldrick JC, Relman DA, Brown PO (2003). Individuality and variation in gene expression patterns in human blood. Proc Natl Acad Sci USA.

[B7] Yang X, Schadt EE, Wang S, Wang H, Arnold AP, Ingram-Drake L, Drake TA, Lusis AJ (2006). Tissue-specific expression and regulation of sexually dimorphic genes in mice. Genome Res.

[B8] Holterhus PM, Hiort O, Demeter J, Brown PO, Brooks JD (2003). Differential gene-expression patterns in genital fibroblasts of normal males and 46, XY females with androgen insensitivity syndrome: evidence for early programming involving the androgen receptor. Genome Biol.

[B9] Chang HY, Chi JT, Dudoit S, Bondre C, van de Rijn M, Botstein D, Brown PO (2002). Diversity, topographic differentiation, and positional memory in human fibroblasts. Proc Natl Acad Sci USA.

[B10] Sinnecker GH, Hiort O, Nitsche EM, Holterhus PM, Kruse K (1997). Functional assessment and clinical classification of androgen sensitivity in patients with mutations of the androgen receptor gene. Eur J Pediatr.

[B11] Thomas PD, Kejariwal A, Campbell MJ, Mi H, Diemer K, Guo N, Ladunga I, Ulitsky-Lazareva B, Muruganujan A, Rabkin S, Vandergriff JA, Doremieux O (2003). PANTHER: a browsable database of gene products organized by biological function, using curated protein family and subfamily classification. Nucleic Acids Res.

[B12] Kresse H, Schonherr E (2001). Proteoglycans of the extracellular matrix and growth control. J Cell Physiol.

[B13] Chiquet-Ehrismann R (2004). Tenascins. Int J Biochem Cell Biol.

[B14] Chanut-Delalande H, Bonod-Bidaud C, Cogne S, Malbouyres M, Ramirez F, Fichard A, Ruggiero F (2004). Development of a functional skin matrix requires deposition of collagen V heterotrimers. Mol Cell Biol.

[B15] Hughes IA, Houk C, Ahmed SF, Lee PA, LWPES Consensus Group; ESPE Consensus Group (2006). Consensus statement on management of intersex disorders. Arch Dis Child.

[B16] The Brown lab. http://cmgm.stanford.edu/pbrown/.

[B17] Tusher VG, Tibshirani R, Chu G (2001). Significance analysis of microarrays applied to the ionizing radiation response. Proc Natl Acad Sci USA.

[B18] Bullinger L, Dohner K, Bair E, Frohling S, Schlenk RF, Tibshirani R, Dohner H, Pollack JR (2004). Use of gene-expression profiling to identify prognostic subclasses in adult acute myeloid leukemia. N Engl J Med.

[B19] Eisen MB, Spellman PT, Brown PO, Botstein D (1998). Cluster analysis and display of genome-wide expression patterns. Proc Natl Acad Sci USA.

[B20] Livak KJ, Schmittgen TD Analysis of relative gene expression data using real-time quantitative PCR and the 2(-delta delta C(T)) method. Methods.

[B21] Elmlinger MW, Mayer I, Schnabel D, Schuett BS, Diesing D, Romalo G, Wollmann HA, Weidemann W, Spindler KD, Ranke MB, Schweikert HU (2001). Decreased expression of IGF-II and its binding protein, IGF-binding protein-2, in genital skin fibroblasts of patients with complete androgen insensitivity syndrome compared with normally virilized males. J Clin Endocrinol Metab.

[B22] Supplemental information. http://microarray-pubs.stanford.edu/AIS-2/.

[B23] Stanford Genomic Resources. http://genome-www.stanford.edu/.

[B24] SOURCE. http://smd-www.stanford.edu/cgi-bin/source/sourceSearch.

